# Prevalence, infant outcomes and gestational risk factors for transverse reduction deficiencies at or above the wrist: a population-based study

**DOI:** 10.1177/17531934241249913

**Published:** 2024-05-23

**Authors:** Ida Neergård Sletten, Jarkko Jokihaara, Kari Klungsøyr

**Affiliations:** 1 Division of Orthopaedic Surgery, Oslo University Hospital, Oslo, Norway; 2 Department of Hand Surgery, Tampere University Hospital, Tampere, Finland; 3 Faculty of Medicine and Health Technology, Tampere University, Tampere, Finland; 4 Division of Mental and Physical Health, Norwegian Institute of Public Health, Bergen, Norway; 5 Department of Global Public Health and Primary Care, University of Bergen, Bergen, Norway

**Keywords:** Congenital upper limb anomaly, infant outcome, limb reduction defect, maternal risk factor, transverse deficiency upper limb

## Abstract

We identified individuals born in Norway between 1970 and 2019 with transverse reduction deficiency at or above the wrist (TRDAW) from the Medical Birth Registry of Norway and from the CULA (congenital upper limb anomaly) North Oslo Registry. Infant outcomes and parental factors were compared for 202 individuals with TRDAW to 2,741,013 living individuals without TRDAW born during the same period. We found an overall TRDAW prevalence of 0.74/10,000. Infants with TRDAW had a higher risk for being small for gestational age, an Apgar score <7 and transfer to neonatal intensive care units after delivery. Nine of the infants with TRDAW had associated anomalies, most commonly in the lower limb, and at a higher proportion than the reference population. Other than twin pregnancies, we are unable to identify with certainty any other risk factors for TRDAW.

**Level of evidence:** I

## Introduction

Transverse reduction deficiency at or above the wrist (TRDAW) is one of the most frequent subtypes of limb reduction defects (Ekblom et al., 2010; Goldfarb et al., 2017; Koskimies et al., 2011; Wynne-Davies and Lamb, 1985) classified as IA1ii or IA1iii in the Oberg-Manske-Tonkin classification of congenital upper limb anomalies (CULA) (Goldfarb et al., 2020). A short arm without a hand is immediately apparent at birth and can be picked up during antenatal ultrasound scanning, causing parental distress. Many have concerns that their child will be severely disabled, and that the anomaly may be associated with other congenital anomalies and syndromes.

The prevalence of transverse limb deficiencies in the whole upper limb (including distal to wrist level) has been reported to be in the range of 0.8–6.8/10,000 (Czeizel et al., 1993; Ekblom et al., 2010; Goldfarb et al., 2017; Koskimies et al., 2011; Wynne-Davies and Lamb, 1985). Upper limb transverse deficiencies have been reported as more common in female infants (Czeizel et al., 1993; Wynne-Davies and Lamb, 1985), usually unilateral and sporadic, and rarely associated with other limb or organ anomalies (Bergman et al., 2020; Czeizel et al., 1993; Wynne-Davies and Lamb, 1985). Others, however, have reported associated anomalies in 25%–28% of patients (Ekblom et al., 2010; Froster and Baird, 1992b; Koskimies et al., 2011).

The pathways for limb development are well-described; there are three axes for morphogenesis, where each axis has its own signalling centre (Oberg, 2019). The proximal-distal axis of limb development is controlled by the apical ectodermal ridge, and it is believed that a disruption in this centre or a disruption of the vascular support of the mesoderm (Bavinck and Weaver, 1986) causes transverse deficiencies. Previous studies have reported young (<25 years) and advanced (>35 years) age (Syvänen et al., 2021), primiparity (Syvänen et al., 2021), smoking (Czeizel et al., 1994), alcohol intake (Froster and Baird, 1992a), thrombophilia (Ordal et al., 2016), misoprostol intake (Gonzalez et al., 1998) and low dietary intake of riboflavin (Robitaille et al., 2009) as maternal risk factors. Maternal pregestational diabetes has also been associated with an increased risk for limb reduction defects in general (Klungsøyr et al., 2019; Syvänen et al., 2021; Zhang et al., 2022).

There is a lack of epidemiological studies focusing on TRDAW. Many studies on limb reduction defects have included longitudinal limb deficiencies, e.g. radial dysplasia, which are known to have a different aetiology (Oberg, 2019) and have a higher association with other anomalies and syndromes (Alberto et al., 2020; Bedard et al., 2018; Bergman et al., 2020; Ekblom et al., 2010; Froster and Baird, 1992; Koskimies et al., 2011; Syvänen et al., 2021; Wynne-Davies and Lamb, 1985). In our clinical experience, TRDAW is rarely associated with other congenital anomalies.

We have previously reported on the prevalence of limb reduction defects from the nationwide, population-based Medical Birth Registry of Norway (MBRN) (Klungsøyr et al., 2019). Due to unspecific details about hand/finger anomalies, we were unable to determine the exact type of all upper limb reduction defects in the MBRN (*n* = 783), although it is possible to identify persons with TRDAW. The primary aim of this study was therefore to report on the Norwegian TRDAW prevalence between 1970 and 2019, and to study the associated epidemiological data including types of pregnancy, delivery and infant outcomes, including other anomalies. The secondary aim was to identify potential risk factors for TRDAW.

## Methods

We conducted the study according to the Strengthening the Reporting of Observational Studies in Epidemiology (STROBE) statement (Vandenbroucke et al., 2007).

### Inclusions from the Medical Birth Registry of Norway

The MBRN was established in 1967 as the world’s first national medical birth registry in the aftermath of the Thalidomide tragedy (Irgens, 2000). Reporting of all births, including live births, stillbirths, spontaneous abortions from 16 gestational weeks (12 weeks from 2002) and termination of pregnancy for fetal anomaly (TOPFA) is mandatory. The registry collects data about maternal age, parity, maternal health status before and during pregnancy, some socioeconomic factors, delivery complications and infant outcomes, including congenital anomalies and mortality. Since 1988, information about assisted reproductive technology (ART) and gestational diabetes has been reported, and since 1999, information about maternal smoking, use of multivitamins and folic acid, ultrasound-based gestational age and infants’ transferal to a neonatal intensive care unit (NICU) after birth. In Norway, all newborns are examined by paediatricians who aim to diagnose and report congenital anomalies. The forms and disease classification systems have changed several times since 1967, but we have reviewed all limb reduction defect reports between 1970 and 1998 and re-coded the anomalies according to the International Classification of Diseases, Tenth Revision (ICD-10) (Klungsøyr et al., 2019). For this study, we included all individuals with the ICD-10 codes Q71.0 (congenital complete absence of upper limb; amelia) and Q71.2 (congenital absence of both forearm and hand). We screened all individuals with the ICD-10 code Q71.3 (congenital absence of hand and finger) because some could have used the Q71.2 code exclusively for a deficiency level at the upper arm or elbow joint, and Q71.3 for the forearm and wrist level. We included individuals with a Q71.3 code if an accompanying free-text description indicated that the whole hand was missing. We did not include individuals with limb defects coded in the MBRN as constriction band syndrome (Q79.80 in the British Paediatric Association Classification, ICD-10 BPA, an extension to the ICD-10).

### Inclusions from the CULA (congenital upper limb anomaly) North Oslo Registry

The registry was established at Oslo University Hospital by one of the authors (INS) in 2018. Patients with TRDAW evaluated since the year 2018 have been included prospectively. Patients with TRDAW evaluated before the year 2018 have been included retrospectively after screening all the electronic medical records from Oslo University Hospital between 1999 and 2018 with all limb reduction defect ICD-10 codes (Q71.0–Q71.9). Any TRDAW diagnosis verified with details from the medical description and/or radiographs, have been (re-)coded in the registry as Q71.0 or Q71.2. As in the MBRN, most individuals with a Q71.3 code in Oslo University Hospital did not have TRDAW. In both registries, the Q71.3 code has been used for transverse deficiencies, both proximal and distal to the wrist, as well as for longitudinal deficiencies (e.g. lack of the thumb in radial longitudinal deficiency and lack of ulnar fingers in ulnar longitudinal deficiency). None of the persons with TRDAW identified in the CULA North Oslo Registry had constriction band syndrome.

### Study population

The Regional Ethical Committee gave permission to include those who did not actively refuse participation after having received a study invitation, i.e. to use their de-identified MBRN data. Therefore, we could not include TOPFA, stillbirths, deceased individuals, or those who had moved abroad or had an unknown postal address. We cross-checked the national identification numbers of all living persons born between 1970 and 2019 with TRDAW from the MBRN and the CULA North Oslo Registry. Both registries are linked with the National Population Register, which contains postal addresses for everyone who resides in Norway. We invited, by mail, all eligible individuals or caregivers to children aged <16 years to participate, and excluded those who replied that they did not have TRDAW (e.g. had a short arm with a wrist and/or fingers), individuals born outside Norway and individuals we were not able to contact. Persons with TRDAW aged above 16 years were also invited to participate in a separate clinical study about arm function, health-related quality of life and prosthesis use, and their outcomes have previously been reported (Sletten et al., 2023).

### Prevalence, gestational risk factors and infant outcomes

We calculated yearly and overall TRDAW prevalence rates and collected MBRN data about pregnancies, deliveries and infant outcomes in participants and the background population of comparable individuals (live-born infants between 1970 and 2019, living in Norway in 2019). The MBRN has incomplete data on uni-/bilateral and right-/left-sided limb reduction defects, and these proportions could not be calculated. We used the European network of population-based registries for the epidemiological Surveillance of Congenital Anomalies classification system to classify other major anomalies (EUROCAT).

### Statistical analysis

We used frequency and contingency tables to describe TRDAW by parental, pregnancy, delivery and infant characteristics. Generalized linear models (GLM) with log link, binomial distribution and exponentiated regression coefficients were used to calculate crude and adjusted prevalence ratios with 95% confidence intervals (CI). When GLM models did not converge, we used Poisson regression with robust standard errors (Zou, 2004). The mother was the unit of analysis, except for operative deliveries (where infants in plural pregnancies could have different deliveries) and infant outcomes (where the infant was the unit of analysis). Analyses where the mother was the unit of analyses were clustered on the mother for correlations between siblings. Maternal age and year of infant birth (five-year groups) were evaluated as possible confounding factors and thus adjusted for.

## Results

**Figure 1.**
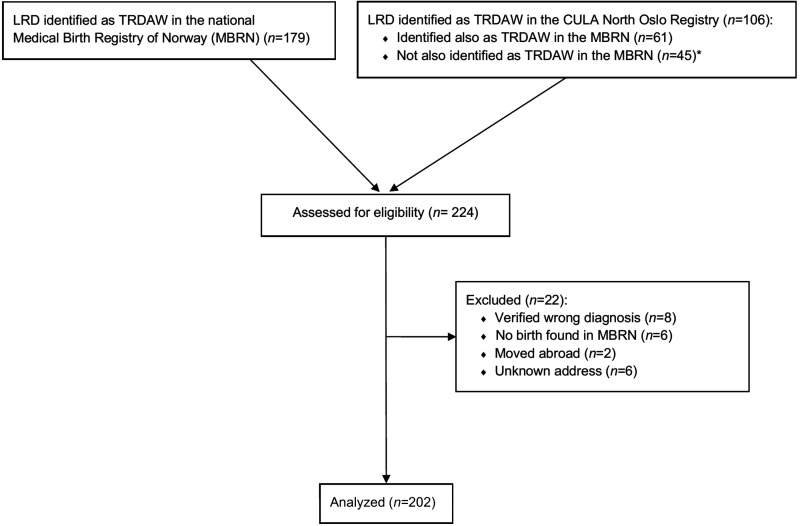
Flow diagram for inclusion and exclusion of participants in the study. *Among the 45 individuals with TRDAW identified solely in the CULA North Oslo Registry, no information of birth was found in the MBRN for six persons who had probably been born outside of Norway. A LRD had been reported to the MBRN for 27 of 39 infants born in Norway, but we were not able to identify it as TRDAW from the combination of anomaly code and text. The LRD had not been reported to the MBRN for the remaining 12 individuals. LRD: limb reduction defect; TRDAW: transverse reduction deficiency at or above the wrist.

From the two registries, we identified 224 eligible persons, of whom 22 were excluded (Figure 1). Out of 118 persons identified only in the MBRN, eight reported that they did not have TRDAW (transverse deficiency distal to the wrist without fingers, *n* = 6; and longitudinal deficiency with a wrist and 1–2 fingers, *n* = 2). MBRN data for 202 individuals (100 male and 102 female) were included in the analyses, as no individuals actively refused to participate.

### Prevalence

**Figure 2.**
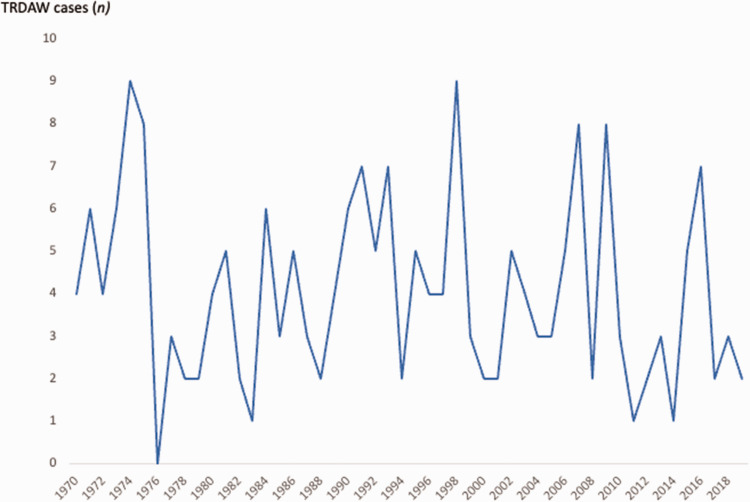
Living individuals with transverse reduction deficiency at or above the wrist (TRDAW) born in Norway between 1970 and 2019.

**Figure 3.**
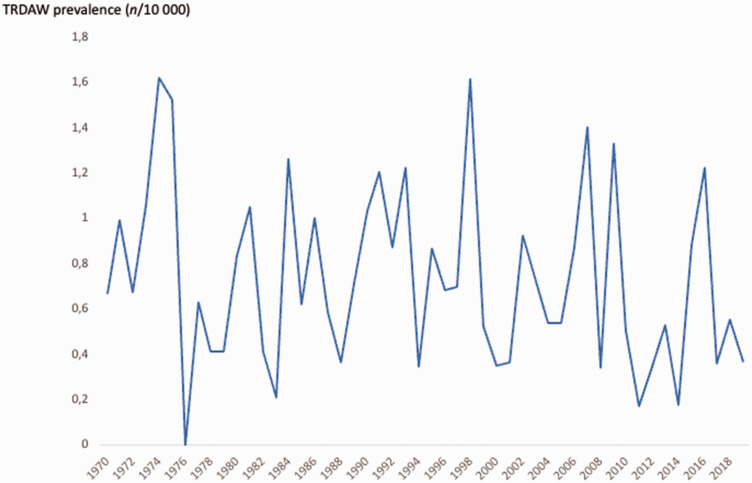
Annual prevalence among living individuals with transverse reduction deficiency at or above the wrist (TRDAW) in Norway between 1970 and 2019.

Between 1970 and 2019, a total of 2,741,216 infants, alive and living in Norway in 2019, were registered in the MBRN, yielding an overall TRDAW prevalence of 0.74/10,000. The number of infants with TRDAW born each year in Norway was in the range of 0–9 (Figure 2), and the prevalence was in the range of 0–1.6/10,000 per year (Figure 3). A total of 10 (5%) individuals had amelia (Q71.0).

### Gestational risk factors and infant outcomes

We could not identify any significant risk factors for TRDAW (Tables 1 and 2); however, there were significantly more twin pregnancies in the TRDAW than in the reference population. We found a twofold risk of being small for gestational age (SGA) both below the 10th and the 2.5th percentile and an almost fourfold risk of Apgar score <7. Infants with TRDAW were three times more frequently transferred to neonatal intensive care units (NICU) after delivery. Among the 25 infants with TRDAW who were transferred to a NICU, we found that one had a 5-minute Apgar score of 6, three had other anomalies outside TRDAW as well as other problems including SGA and respiratory problems, three were preterm, two had sepsis, one had respiratory problems and hypoglycaemia, three had icterus, one had intrauterine hypoxia and hematologic disturbances, and two were large for gestational age. The remaining nine infants were transferred only for a closer check-up because of their TRDAW.

**Table 1.** Maternal demographics and prepregnant chronic conditions in mothers giving birth to 202 infants with congenital TRDAW compared to mothers giving birth to infants without this defect (*n* = 2,703,777) in Norway between 1970 and 2019.

	TRDAW	No TRDAW	Crude PR (95% CI)	Adjusted PR^a^ (95% CI)
Maternal demographics				
Age ≥35 years	25 (12.4)	342,996 (12.7)	0.98 (0.68–1.41)	1.01 (0.71–1.46)
Age <20 years	9 (4.5)	121,890 (4.5)	0.99 (0.52–1.87)	0.94 (0.50–1.75)
Single partner status	12 (5.9)	255,587 (9.5)	0.63 (0.36–1.09)	0.64 (0.38–1.09)
Nulliparous	90 (44.6)	1,127,743 (41.7)	1.07 (0.92–1.25)	1.07 (0.92–1.23)
Paternal demographics^b^				
Age ≥40 years	20 (10.1)	248,690 (9.3)	1.09 (0.72–1.65)	1.12 (0.74–1.69)
Age <20 years	3 (1.5)	28,042 (1.0)	1.45 (0.47–4.46)	1.39 (0.46–4.21)
Maternal prepregnant disease				
Pregestational diabetes	1 (0.5)	11,754 (0.4)	1.14 (0.16–8.04)	1.20 (0.17–8.41)
Chronic hypertension	1 (0.5)	9,874 (0.4)	1.36 (0.19–9.58)	1.42 (0.20–10.0)
Chronic kidney disease	2 (1.0)	24,184 (0.9)	1.11 (0.28–4.40)	1.10 (0.28–4.37)
Rheumatic arthritis	1 (0.5)	8,521 (0.3)	1.57 (0.22–11.10)	1.59 (0.22–11.28)
Any chronic disease^c^	13 (6.4)	145,289 (5.4)	1.20 (0.71–2.03)	1.24 (0.74–2.09)

Data are expressed as n (%) unless otherwise indicated.

a Adjusted for period of birth (5-year categories) and maternal age (<20, 20–24, 25–29 [ref], 30–34, 35–39, 40+ years).

b Information on fathers available for 99.2% of pregnancies in the reference population.

c Asthma, chronic hypertension, chronic renal disease, rheumatic arthritis, pregestational diabetes, epilepsy.

CI: confidence interval; PR, prevalence ratio; TRDAW, transverse deficiency at or above the wrist level.

**Table 2.** Pregnancy, delivery and infant outcomes for mothers and their 202 infants with congenital TRDAW compared to mothers (*n* = 2,703,777) and their infants (*n* = 2,741,014) without this defect in Norway between 1970 and 2019.

	TRDAW	No TRDAW	Crude RR (95% CI)	Adjusted RR^a^ (95% CI)
Pregnancy				
Maternal smoking^b^	12 (20.7)	132,503 (13.8)	1.50 (0.90–2.48)	1.53 (0.95–2.46)
Use of folate supplements^b^	39 (60.9)	697,367 (71.0)	0.86 (0.71–1.04)	0.85 (0.71–1.02)
ART pregnancy^c^	5 (3.9)	38,835 (2.2)	1.78 (0.76–4.21)	1.98 (0.85–4.61)
Multiple pregnancy	6 (3.0)	36,528 (1.4)	2.20 (1.00–4.83)	2.25 (1.02–4.93)
Gestational diabetes^c^	3 (2.3)	29,947 (1.7)	1.39 (0.45–4.25)	1.56 (0.51–4.76)
Pre-eclampsia	7 (3.5)	83,074 (3.1)	1.13 (0.54–2.34)	1.13 (0.55–2.35)
Pregnancy hypertension	6 (3.0)	44,378 (1.6)	1.81 (0.82–3.98)	1.85 (0.84–4.05)
Delivery				
Placental abruption	1 (0.5)	11,038 (0.4)	1.21 (0.17–8.57)	1.18 (0.17–8.39)
Excessive bleeding	13 (6.4)	301,823 (11.2)	0.58 (0.34–0.98)	0.63 (0.37–1.07)
CS, total	24 (11.9)	333,467 (12.2)	0.98 (0.67–1.42)	1.01 (0.70–1.46)
CS, elective	9 (4.8)	101,608 (4.1)	1.19 (0.63–2.25)	1.23 (0.66–2.30)
Vaginal instrumental	15 (8.4)	209,928 (8.7)	0.97 (0.60–1.57)	1.00 (0.62–1.63)
Infant outcome				
Preterm	11 (5.8)	150,787 (5.7)	1.01 (0.57–1.79)	1.02 (0.57–1.80)
Very preterm	2 (1.0)	18,179 (0.7)	1.52 (0.39–6.04)	1.56 (0.39–6.19)
LBW	12 (5.9)	116,494 (4.3)	1.40 (0.81–2.42)	1.40 (0.81–2.43)
SGA 10	39 (19.3)	270,863 (9.9)	1.95 (1.47–2.59)	1.93 (1.46–2.56)
SGA 2.5	11 (5.5)	69,892 (2.6)	2.14 (1.20–3.79)	2.08 (1.17–3.68)
Apgar score <7	6 (3.0)	23,603 (0.9)	3.45 (1.57–7.59)	3.64 (1.66–7.99)
Apgar score <4	1 (0.5)	3873 (0.1)	3.51 (0.50–24.75)	3.71 (0.52–26.28)
Anomalies outside TRDAW	9 (4.5)	25,583 (0.9)	4.77 (2.52–9.04)	5.41 (2.80–10.46)
Transferred to NICU^b^	25 (34.7)	128,205 (11.2)	3.10 (2.26–4.25)	3.11 (2.27–4.25)

Data are expressed as *n* (%) unless otherwise indicated. The mother is the unit of analysis when analysing pregnancy factors, abruptio and bleeding, and the infant when analysing instrumental vaginal delivery, caesarean section and infant outcomes.

a Adjusted for period of birth (5-year categories) and maternal age (<20, 20–24, 25–29 [ref], 30–34, 35–39, 40+ years).

b Available from 1999.

c Available from 1988.

ART: assisted reproductive technology; CI: confidence interval; CS: caesarean section; LBW: low body weight (<2500 g); NICU: neonatal intensive care unit; RR: risk ratio; SGA 2.5: small for gestational age below the 2.5th percentile; SGA 10: small for gestational age below the 10th percentile; TRDAW: transverse deficiency at or above the wrist level.

In total, 9 (4.5%) of the infants with TRDAW had additional congenital anomalies, compared to 25,583 (0.9%) in the reference population (Table 2). The additional anomalies for two infants with amelia (Q71.0) were microgastria (*n* = 1) and complete absence of lower limb (*n* = 1). For seven infants with a more distal TRDAW, the associated anomalies were: Down syndrome (*n* = 1); Möbius syndrome (*n* = 1); cleft lip/palate (*n* = 2); absence of foot and/or toes (*n* = 2); and toe syndactyly (*n* = 1).

Our study population included only individuals who were alive and living in Norway at the start of the study. In data from the MBRN, we found a total of 29 TRDAW stillborn or TOPFA cases and five deceased individuals with TRDAW, three of these within the first 28 days of life. Of these 34 cases, 25 (74%) had other congenital anomalies.

## Discussion

We conducted a national, epidemiologic study of individuals with TRDAW born in Norway between 1970 and 2019 and still alive in the year 2019, and we analysed gestational risk factors and infant outcomes. We found an overall TRDAW prevalence of 0.74/10,000. We also identified certain associations with TRDAW, such as being SGA and moderately reduced Apgar scores.

The variation in the Norwegian annual TRDAW prevalence was small, and our overall prevalence was in accordance with the prevalence of 0.8–1.3/10,000 in four previous studies that included transverse deficiencies distal to the wrist (Czeizel et al., 1993; Ekblom et al., 2010; Goldfarb et al., 2017; Koskimies et al., 2011). A Scottish study reported a 10-fold prevalence (6.8/10,000), but the background population was not described well enough for further review (Wynne-Davies and Lamb, 1985). The female to male ratio was 1:1 in our study, in contrast to our previous finding of a higher risk for male offspring in all limb reduction defects (Klungsøyr et al., 2019), and also in contrast to the findings of more female infants with whole upper limb transverse deficiencies in previous studies (Czeizel et al., 1993; Wynne-Davies and Lamb, 1985).

Infant data specifically for TRDAW has not been previously reported. Our data give no explanation for the higher risk of a moderately reduced Apgar score. Our finding of a higher risk for SGA may be explained by the lesser weight due to a shorter or missing arm. In children without birth defects, SGA is usually a result of intrauterine growth restriction, and factors such as smoking or pregnancy hypertension/pre-eclampsia. However, in our population, the prevalence of smoking, hypertension and pre-eclampsia in mothers to infants with TRDAW was not significantly higher than in mothers to infants without TRDAW. However, numbers were low, and we cannot entirely exclude the possibility of maternal and/or placental reasons playing an additional role. The higher transferal rate to the NICU after delivery could also be partly explained by a more meticulous follow-up due to the finding of TRDAW.

We found further anomalies in individuals with TRDAW than in the reference group, but the proportion was less than one-tenth of what was found for all cases of limb reduction defects in Norway between 1999 and 2016, including longitudinal deficiencies and deficiencies found in stillbirths and TOPFA (Klungsøyr et al., 2019). Cardiac anomalies were the most common in all cases of limb reduction defects (Klungsøyr et al., 2019), but these were not found among individuals with TRDAW in the present study. We found one case of TRDAW with Möbius syndrome, a syndrome known to be associated with symbrachydactyly (Koehler et al., 2019). We found no individuals with Poland syndrome, and believe that associated anomalies reported for children with transverse deficiencies across different studies are mainly due to the inclusion of deficiencies distal to the wrist (Alberto et al., 2020; Bedard et al., 2018; Bergman et al., 2020; Czeizel et al., 1993; Ekblom et al., 2010; Koskimies et al., 2011). Differences in the study populations could also play a role (including or excluding deaths, stillbirths and TOPFAs). A Dutch study found that none of the individuals with an isolated limb reduction defect had a genetic disorder (Bergman et al., 2020). Out of 202 individuals in our cohort, only one had an associated anomaly not immediately detectable on clinical examination (microgastria). However, our population included those who survived infancy and the number was perhaps too small for us to conclude whether postpartum radiological screening of internal organs or referral to a geneticist is indicated.

We found a higher risk for TRDAW in twin pregnancies but are unaware of any aetiological explanation. Previously reported associations between transverse deficiencies and maternal smoking, thrombophilia, and intake of alcohol and misoprostol, and the higher frequency of left-sided defects have suggested a vascular theory for apical ectodermal ridge signalling centre disruption (Czeizel et al., 1994; Froster and Baird, 1992a; Gonzalez et al., 1998; Ordal et al., 2016). Of these suggested risk factors, we only had MBRN data about smoking from the year 1999. Among 74 TRDAW pregnancies in this period, smoking information was registered for 58 mothers, and we found no association between maternal smoking and TRDAW. However, from the adjusted risk ratios and 95% CIs, we can suspect associations between both smoking and low folate intake and TRDAW. We have previously found folate supplements to be associated with a significantly lower risk of limb reduction defects (Klungsøyr et al., 2019), in accordance with several other studies (Canfield et al., 2005; Shaw et al., 1995; Werler et al., 1999; Wolf et al., 2017); however, this remains a debatable point (Bower et al., 2006; Robitaille et al., 2009).

We previously found a higher risk for limb reduction defects in nulliparous mothers and mothers with pregestational diabetes (Klungsøyr et al., 2019), which was not found in the present study. A Finnish case-control study that included live births, stillbirths and fetuses from spontaneous abortions and TOPFA also found a higher risk for limb reduction defects associated with nulliparity, pregestational diabetes and, in addition, for mothers who used antiepileptic drugs (Syvänen et al., 2021). They also found a higher risk for transverse deficiencies in young (<25 years) and advanced (>35 years) maternal age, primiparity, use of topical anti-infective gynaecological drugs and use of tetracyclines, but the uncertainty was high for the latter two outcomes.

Larger population-based studies from multiple countries are recommended to investigate risk factors further, but the lack of an unambiguous classification system hinders conclusions to be drawn for subtypes that do not share the same aetiology. The nomenclature for describing CULA has evolved since the 1970s. In a recent study of historical case notes archived in a tertiary congenital hand and amputee clinic, the majority of diagnoses given in the 1960s and the 1970s differed from today’s re-classification according to the Oberg-Manske-Tonkin (OMT) classification (Chan et al., 2023). Another challenge is the use of different classification systems among physicians from different medical specialities. Epidemiologists use the ICD-10 and the ICD-10 BPA, while congenital hand surgeons are recommended to use the OMT classification. The spectrum of transverse limb defects is subdivided into symbrachydactyly and transverse deficiencies in the OMT, but the inter-rater reliability of distinguishing between these two phenotypes has been found to be low (Sletten et al., 2022). Furthermore, imprecise descriptions, e.g. in the ICD-10 classification, may seem unimportant in clinical practice but are of utmost importance in epidemiological studies where identifying causal relations are an important aim and the conclusions are, eventually, based on clinicians’ routine descriptions and inclusion in national registries. In our opinion, transverse deficiencies should be classified in the ICD-10 precisely as the level of the most distal part of the limb (stump ending at the level of the shoulder, arm, elbow, forearm, wrist, metacarpals or phalanges) rather than today’s ambiguous description of what limb parts are missing. Ideally, physicians from all medical specialities would use an unambiguous identical classification system to strengthen interdisciplinary cooperation in epidemiological studies on CULA.

This study is the largest epidemiological study on TRDAW so far. It is based on a birth registry with mandatory notification covering an entire country, where all cases of limb reduction defects since 1970 have been cross-checked and recoded to ICD-10. From 1999, cases were also included from a clinical CULA registry, where all cases were verified with details from the medical descriptions and/or radiographs. The main limitation is the small number of included individuals due to TRDAW being a rare anomaly. We had 50 years of data to calculate estimates of infant outcomes, but associated anomalies were more poorly registered before 1999 (Klungsøyr et al., 2019). Norway is a small country, and potential maternal risk factors were generally rare. However, the exclusion of stillbirths, TOPFA cases and infants who died might have influenced our risk analyses. There is uncertainty if all eligible individuals were identified, as we found 39 patients with TRDAW born in Norway in the CULA North Oslo Registry who were not identifiable as having TRDAW from the MBRN. For 27 of these, the limb reduction defect had been reported to the MBRN, but it was not possible to verify as TRDAW from the combination of ICD-10 code and eventual free text. In addition, more than the eight excluded persons who reported a wrong diagnosis might have been misclassified. Despite this potential misclassification bias, we have no reason to believe that our material suffered selection bias, and we do not fear that it affected our main findings.

In conclusion, we found a TRDAW prevalence that was in accordance with reports from other countries. Persons with TRDAW had a higher risk of SGA, reduced Apgar score and transferal to NICU compared to those without TRDAW. The proportion of persons with other anomalies was higher than in the reference population, but much lower than for limb defects in general. We could not with certainty identify parental or pregnancy risk factors other than twin pregnancy. This study provides information that are potentially helpful for obstetricians, paediatricians, geneticists and hand surgeons who can better inform parents pre- and postnatally.
